# Odorants in Fish Feeds: A Potential Source of Malodors in Aquaculture

**DOI:** 10.3389/fchem.2018.00241

**Published:** 2018-06-25

**Authors:** Mohamed A. A. Mahmoud, Thorsten Tybussek, Helene M. Loos, Maria Wagenstaller, Andrea Buettner

**Affiliations:** ^1^Chair of Aroma and Smell Research, Department of Chemistry and Pharmacy, Emil Fischer Center, Friedrich-Alexander-Universität Erlangen-Nürnberg, Erlangen, Germany; ^2^Sensory Analytics Department, Fraunhofer Institute for Process Engineering and Packaging IVV, Freising, Germany; ^3^Agricultural Biochemistry Department, Faculty of Agriculture, Ain Shams University, Cairo, Egypt; ^4^Department of Retention of Food Quality, Fraunhofer Institute for Process Engineering and Packaging IVV, Freising, Germany

**Keywords:** fish feed, aquaculture, SAFE, cAEDA, GC-O, off-flavor

## Abstract

Although the microbiota is considered to be the primary source of off-flavors in farmed fish, there is a lack of information about the possible contribution of feeds to fish malodor. For this reason, the current study was designed to perform comprehensive sensory and chemo-analytical characterization of fish feed constituents that can impact the quality of farmed fish, and to determine whether feeds cause malodor accumulation in fish. To this aim, odorants in four commercial fish feeds were extracted using solvent assisted flavor evaporation (SAFE) and characterized by comparative aroma extract dilution analysis (cAEDA) and multi-dimensional gas chromatography-mass spectrometry/olfactometry (MD-GC-MS/O). The odorants in the fish feed samples were correlated with their respective sensory and fatty acid profiles. The cAEDA studies revealed the presence of 81 odorants of which 55 compounds were common to all the samples. Most of these odorants are identified here for the first time in fish feeds, and include skatole, indole, (*E,Z,Z*)-2,4,7-tridecatrienal, 4-ethyloctanoic acid, and cresols. Additionally, geosmin and 3-isopropyl-2-methoxypyrazine, known for their contribution to fish taint, and other cyanobacterial by-products, dimethyldisulfide and dimethyltrisulfide, were identified in feed samples. The results suggest that fish feed may contribute to fish malodor. Most of these off-flavors were linked to lipid source (fish oil or plant/lard alternatives), unsaturated fatty acids contents, and protein type (plant-based or fishmeal-based sources) in the feed.

## Introduction

In recent years aquaculture has been growing at an annual rate of 3.2%. This is mostly attributed to developments in fish feed manufacture, namely the biggest cost factor, and the use of more productive aquaculture systems (FAO, 2016).

The main aims of fish feed manufacturers are: (I) to lower production costs, (II) to increase feed conversion rates by the fish, and (III) to decrease feed waste (Olsen, 2011). The attainment of these aims, however, does not necessarily guarantee that fish with high palatability are produced.

Lipids and proteins are the major ingredients of fish feed (Olsen, 2011; Shen et al., 2018). Odor-active compounds are known to originate from these constituents during the fish feed manufacturing process. These compounds might affect the final quality and consumer acceptability of fish. For example, lipid sources rich in polyunsaturated fatty acids (PUFA) can result in elevated levels of PUFA-derived volatile aldehydes during feed processing that may result in off-flavor formation in fish fed on these feeds (Turchini et al., 2007). Additionally, several pyrazines, having a wide range of odors from roasted-like to distinctive earthy, can arise due to the thermal treatment during feed pellet formation (Mjøs and Solvang, 2006; Mahmoud and Buettner, 2017).

In many cases, the raw material itself already contains elevated levels of off-flavors even before undergoing the feed processing procedure. For example, using pig lard to entirely or partially replace fish oil in feed formulae can lead to accumulation of fecal-like and sweat-like smelling compounds skatole and androstenone in fish after feed consumption (Zhou et al., 2015; Mahmoud and Buettner, 2016). Moreover, compounds with blood-like and metallic odors are increased by using blood as a raw material for feed production (Aladetohun and Sogbesan, 2013; Nilsson et al., 2014). All this evidence means that fish feeds must be considered as potential sources of malodors in fish. Indeed, odorants originating from the feed can directly accumulate in fish after consumption (Howgate, 2004). On the other hand, odorants from unconsumed feed can disperse in the water and later accumulate in fish via respiration or through the skin, potentially even after further modification via reactions taking place in the aqueous medium (Howgate, 2004; Podduturi et al., 2017).

The volatile composition of fish feeds has been investigated in several studies. However, feeds have only been discussed as a potential source of off-odors in a few reports (Turchini et al., 2004; Giogios et al., 2009; Grigorakis et al., 2009; Alexi et al., 2016; Podduturi et al., 2017). None of these studies used methods that distinguish between odor-active and odorless compounds such as gas chromatography-olfactometry (GC–O). Furthermore, they did not correlate the odor contents with the sensory profiles of the feeds. However, Podduturi et al. (2017) performed a study on fish feeds and suspected that terpenes from fish feed might cause malodors in cultured fish. In their experiment, they tentatively identified and semi-quantified terpenes in fish meat and compared the results with those from the relevant water and feeds using dynamic headspace gas chromatography-mass spectrometry (DHS–GC–MS). Whenever the concentration of an identified terpene was above its published odor threshold it was considered to cause fish malodors. The authors confirmed that fish feeds were the primary source of terpenes in fish meat. However, they stated that their work was incomplete, as they relied on published odor threshold data and most of the identified terpenes had no recorded threshold values in the literature. This limitation can easily be overcome by using odor intensity measuring methods such as aroma extract dilution analysis (AEDA) followed by quantification using stable isotope dilution analysis (SIDA) and aroma reconstitution experiments (Buettner and Schieberle, 2001; Grosch, 2001; Mahmoud and Buettner, 2017).

Our previous studies reported the aquaculture water odor composition and the potential link between the quality of the water and the respective fish aroma (Mahmoud and Buettner, 2016, 2017). A series of odorants were reported for the first time in cultured fish including, amongst others, 3-methylindole (skatole; odor quality: fecal), 5α-androst-16-en-3-one (androstenone; odor quality: sweat-like), 4-ethyloctanoic acid (odor quality: goat-like), and rotundone (odor quality: black pepper). These odorants were identified using established analytical methods, namely one- and two-dimensional gas chromatography-mass spectrometry/olfactometry (1D- and 2D-HRGC-MS/O) and sensory analysis. Several suggestions were put forward to explain the sources of these compounds. However, further studies are required to resolve the origins and formation or accumulation pathways of such potent odorants in aquaculture. Only then targeted avoidance strategies can be elaborated to reduce the accumulation of malodors in fish, hence to improve its sensory quality.

One step in this direction is to study the correlation between the lipid and fatty acid contents of different fish feeds and their aroma profiles and odorant compositions to determine whether they cause malodor accumulation. Accordingly, the current study aimed to perform comprehensive sensory and chemo-analytical characterization of active constituents that can impact fish quality in aquaculture.

## Materials and methods

### Chemicals

Reference compounds and suppliers were as follows: dichloromethane (Merck, Darmstadt, Germany), 3-methylbutanoic acid ≥ 99%, dimethyl trisulfide (DMTS) ≥ 98%, 2-ethyl-3,5-dimethylpyrazine ≥ 95%, 2-ethyl-3,6-dimethylpyrazine ≥ 95%, 2,3,5-trimethylpyrazine ≥ 99%, hexanal ≥ 98%, 1-octen-3-one ≥ 50%, octanal ≥ 99%, 3-hydroxy-4,5-dimethyl-2(5*H*)-furanone (sotolone) ≥ 97 %, (*E)*-2-nonenal ≥ 97%, 3-isobutyl-2-methoxypyrazine ≥ 97%, *(Z*)-2-nonenal ≥ 95%, 2-methylisoborneol (MIB) ≥ 95%, 4-ethyloctanoic acid ≥ 98%, 3-methylindole (skatole) ≥ 98%, γ-decalactone ≥ 98%, γ-nonalactone ≥ 98%, β-ionone ≥97%, 5α-androst-16-en-3-one (androstenone), geosmin ≥ 98%, decanoic acid ≥ 98%, hexanoic acid ≥ 99.5%, decanal ≥ 92%, nonanoic acid ≥ 97%, (*E,E*)-2,4-decadienal ≥ 85%, 1,2-benzopyranone (coumarin) ≥ 99%, dodecanoic acid ≥ 98%, hexadecan-1-ol ≥ 99, hexadecanoic acid 99%, hexadecanoic acid ethyl ester >99%, 2-methylhexanoic acid ≥ 99%, (*E,E*)-2,4-decadienal ≥ 85%, 8-heptadecene ≥ 96.0%, hexadecanoic acid ethyl ester >99%, caryophyllene 80%, 2-phenoxyethanol 99%, phenylacetic acid 99%, α-terpineol 96%, (*E,Z*)-2,6-nonadienal 95%, (*E,E*)-2,6-nonadienal 95%, γ-terpinene 97%, D-limonene 97%, 3-(methylthio)-propanal (methional) 96%, heptanal >92%, 3-isopropyl-2-methoxypyrazine 97% (IPMP), (*Z*)-4-heptenal 98% (Aldrich, Steinheim, Germany), butane-2,3-dione ≥ 99%, nonanal ≥ 95%, butanoic acid ≥ 99.5%, indole 98.5%, 2-ethylhexan-1-ol >99%, 1-octen-3-ol 98%, pentanoic acid 99%, dimethyl disulfide (DMDS) ≥ 98% (Fluka, Steinheim, Germany), (*Z)*-3-hexenal ≥ 50%, γ-dodecalactone ≥ 97%, 4-ethylvanillin 88%, (SAFC, Steinheim, Germany), 4-hydroxy-3-methoxybenzaldehyde (vanillin) ≥ 99% (ABCR, Karlsruhe, Germany), (*E*)-4,5-epoxy-(*E*)-2-decenal ≥ 97%, γ-(*Z*)-6-dodecenolactone, (*Z*)-1,5-octadien-3-one 99% (aromaLAB AG, Munich, Germany), (*E,Z,Z)-*2,4,7-tridecatrienal (kindly provided by Nestle, Switzerland), rotundone (kindly provided by Symrise, Germany).

### Samples

Four commercial fish feeds (coded by with us with the labeled S-1, S-2, S-3, and S-4) were provided to us by local aquaculture farmers and were chosen because they are the most commonly used feeds for salmonid fish not only in Bavaria State, Germany, but also worldwide. One more selection criterion was that all four feeds are produced using extrusion technology. They were directly taken from sealed packages. The samples were directly transported to our institute and stored in a well-ventilated room at 21°C. The pellets were powdered in a lab scale grinder prior to further analysis, except for the sensory test where samples were investigated as a whole.

### Sensory analysis

Sensory analyses were done in a well-lit and ventilated sensory room. The tests were carried out at room temperature. Samples were coded with a random three-digit number before presenting them in covered glass vessels. 13 trained panelists (10 females, and 3 males, mean age: 31 years, range: 24 to 55 years), with no known illness at the time of the experiment, participated in the sensory sessions. They were recruited from the Fraunhofer IVV sensory expert panel (Freising, Germany). All panelists were experienced in GC-O analysis.

The assessment was done in one session consisting of three parts. In the first part, the panelists were asked to orthonasally evaluate all samples individually and to establish a list of sensory attributes which described the samples best. In the second part, they jointly defined the characteristic qualities. Finally, each panelist scored the intensities of attributes on a scale from 0 (no perception) to 10 (strong perception). Additionally, they were asked to evaluate the overall odor intensities on the same scale.

### Lipid analysis

#### Lipid extraction

Lipids were extracted at room temperature from 111 ± 11 mg of powdered samples using 20 mL of *n*-hexane in the presence of 12 ± 2 mg of internal standard (heptadecanoic acid; 17:0). The mixtures were then shaken for one and a half hour. One mL of each solution was collected in 2 mL GC vials, and the *n*-hexane was evaporated using a gentle stream of N_2_. For esterification, 1 mL of *tert*-butylmethylether and 300 μL of 0.2 M methanolic trimethylsulfoniumhydroxide solution were added to the vials before they were sealed and heated at 100°C for 20 min.

#### Gas chromatography with flame ionization detector (GC-FID)

The measurements were performed using an Agilent 7890A gas chromatograph (Agilent Technologies, USA) equipped with a split-/splitless injector and flame ionization detector (FID). Separation was performed using ZB-FFAP column (15 m × 0.25 mm, 0.25 μm; Phenomenex, USA). 1 μL of the sample was injected at 250°C with a split ratio of 1:20, and the carrier gas flow was 11.29 mL min^−1^.

The initial column temperature was 160°C and was held for 1.5 min. Then, the temperature was raised to 250°C with a rate of 37°C min^−1^ and held for 2.5 min. The temperature of FID was set to 260°C. OpenLab C.01.02 with integrated Maestro 1.1 software (Gerstel, Germany) was applied for data acquisition and processing.

### Odorant analysis

#### Odorant enrichment

Odorants were extracted at room temperature from 5 ± 0.2 g of powdered samples using 20 mL of dichloromethane (DCM). After 30 min of stirring the mixture, DCM phases were separately collected and dried over anhydrous Na_2_SO_4_ and distilled using solvent assisted flavor evaporation (SAFE) according to Engel et al. (1999). Finally, the volume was reduced to approximately 100 μL via Vigreux distillation at 50°C (Bemelmans, 1979).

#### One-dimensional high-resolution gas chromatography/olfactometry coupled with flame ionization detection or mass spectrometry (HRGC-FID/O and HRGC-MS/O)

The analyses were performed by means of a helium GC (Thermo Finnigan, Dreieich, Germany) using DB-FFAP (30 m × 0.32 mm fused silica capillary, 0.25 μm; Agilent J&W GC Columns, USA) and DB-5 (30 m × 0.32 mm fused silica capillary, 0.25 μm; Agilent J&W GC Columns, USA). The injection volume was 2 μL and the carrier gas flow was 2.2 mL min^−1^. The initial temperature for both columns was 40°C and was held for 2 min. Then, the temperature was raised at 8°C min^−1^ till 250°C and held for 10 min (DB-5), or to 245°C and held for 8 min (DB-FFAP). The temperature of both sniffing port and FID was set to 270°C. The GC effluent was split 1:1 between the sniffing port and the detector (FID or MS; DSQ, Thermo Finnigan, Dreieich, Germany). The linear retention index (RI) of each compound was calculated according to (Den Dool and Kratz, 1963). Mass spectra were obtained in the electron impact (EI) mode using the following conditions: 70 eV ionization energy, mass range 35 to 249 m/z, scan rate 500 amu/s and a source temperature of 200°C. Three experts were recruited from the sensory panel of Fraunhofer IVV to perform the GC-O analyses. They had experience in recognizing the odor-active compounds in matrices similar to fish feed, i.e., aquaculture water and fish samples from our previous studies.

#### Two-dimensional high-resolution gas chromatography-mass spectrometry/olfactometry (2D-HRGC-MS/O)

The 2D-HRGC-MS/O system consisted of two CP 3800 GCs (Varian, Darmstadt, Germany) coupled with a Saturn 2200 MS (Varian, Darmstadt, Germany). Separation of the volatile substances was performed using the same capillaries as in HRGC-O. The initial temperature (40°C) was held for 2 min, then raised at 6°C min^−1^ to 240°C and held for 5 min in the first oven (DB-FFAP), and at 10°C min^−1^ to 250°C and held for 5 min in the second one (DB-5). The flow rate of the helium carrier gas was 2.5 mL min^−1^. At the end of the capillary, the effluent was split to an olfactory detection port (ODP; Gerstel, Mühlheim, Germany) and an FID (first oven), or to an ODP and an MS (second oven). The FID and the sniffing port were held at 300°C and 270°C, respectively. MS conditions were the same as in the DSQ system.

The identification of odorants was based on the following parameters: retention indices on two columns of different polarity (DB-FFAP and DB-5) and the respective mass spectra obtained in EI mode. Mass spectrometric data obtained for the target compounds were compared with those of the Fraunhofer IVV-internal library and finally confirmed by the respective chemical reference standards in each case. The automated mass spectral deconvolution and identification system software (AMDIS, 32) was used to analyze the full scan mode data. Using AMDIS helped in overcoming sample complexity and matrix interference.

#### Comparative aroma extract dilution analysis (cAEDA)

The flavor dilution factors (FD) of the main odorants were determined as described by Buettner and Schieberle (2001) and Grosch (2001). The concentrated odor distillates (*FD* = 1; 100 μL) were diluted stepwise (1:2; v/v) using DCM, and 2 μL of the dilutions corresponding to FD 3 to 2187 were applied for GC-O analysis on FFAP column. The FD factor for odorants represent the last dilution in which each of the substances was still perceived.

### Ethics statement

The sensory experiment was conducted in agreement with the Declaration of Helsinki. The Ethical Committee of Friedrich-Alexander Universität Erlangen-Nürnberg's Medical Faculty stated that no ethics approval was required as an expert panel carried out the evaluation.

### Statistical analysis

The results obtained from sensory analyses were tested for outliers (Gruber test) and normal distribution (Jarque-Bera test) using XLSTAT 2017® (Addinsoft, Paris, France). Then, they were averaged and plotted in spider-web diagrams using Excel 2016®. One-way analysis of variance (ANOVA) followed by Tukey test (a *p*-value < 0.05 was considered significant) was carried out. For the principal component analysis (PCA), the data (intensity ratings of sensory attributes, FD factors of odorants, total fat, and fatty acid content) were standardized using the z-score; then a correlation test (Pearson correlation) was conducted between the intensity ratings of sensory attributes and the other variables. Both the sensory data and its significantly correlated chemical variables were selected for running the PCA using XLSTAT 2017® (Addinsoft, Paris, France).

## Results and discussion

### Sensory evaluation

**Figure 1:**
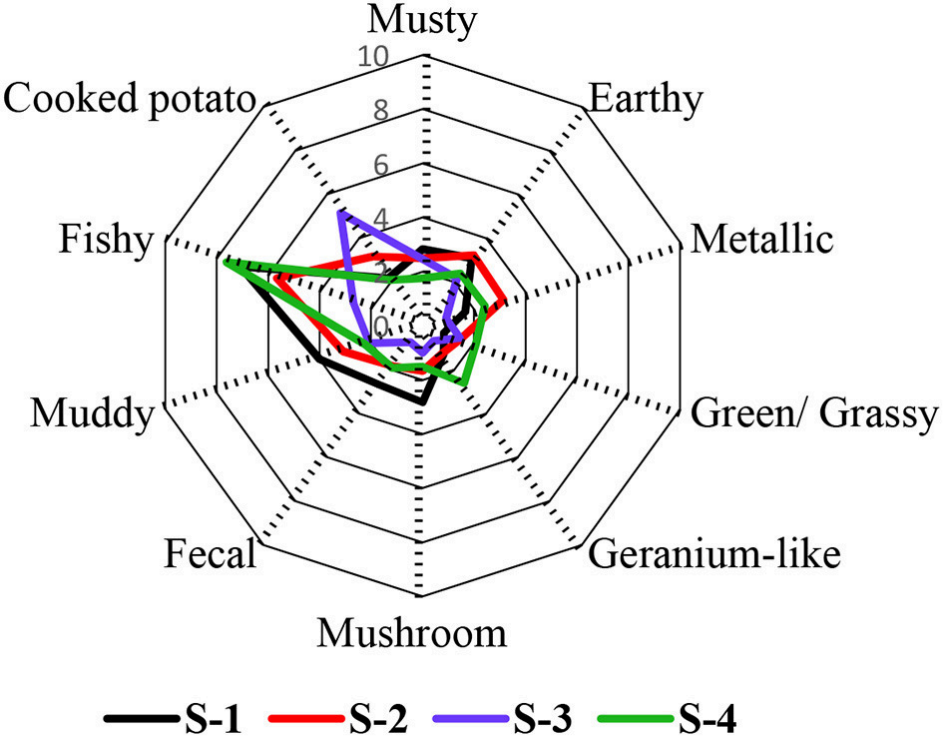
Odor sensory profile analysis of four commercial fish feeds. The data are displayed as mean values of the ortho-nasal sensory evaluation of three independent replicates (13 panelists).

Ten attributes were chosen to describe the smell of the four fish feed samples S-1, S-2, S-3, and S-4. These were the attributes musty, earthy, metallic, green/ grassy, geranium-like, mushroom-like, fecal, muddy, fishy, and cooked potato-like. Based on the intensity ratings, the samples were found to be statistically different (*p* < 0.05). The fishy odor impression was dominant in the feeds S-1, S-2, and S-4 with intensities of 7.1, 5.7, and 7.6 respectively, while it was the second highest attribute in S-3, with an intensity score of 2.7 (Figure 1). The fishy smell was therefore one of the leading contributors to the overall aroma of these samples. In the case of S-3, however, the odor attribute with the highest intensity was cooked potato-like. Regarding the four different feeds, the highest rated smell attributes in S-1 were fecal, mushroom-like, musty, and muddy, whereas the metallic and earthy impressions were ranked highest in S-2, cooked potato-like was rated highest in S-3, while green/grassy, geranium-like, and fishy attributes were rated highest in S-4.

**Figure 2:**
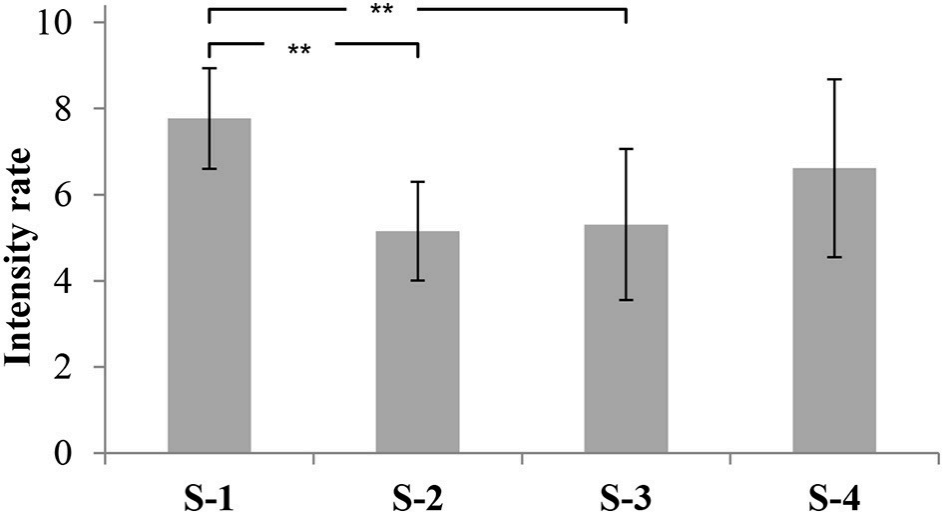
Overall odor intensities of four commercial fish feeds. The data are displayed as mean values of the ortho-nasal sensory evaluation (13 panelists) of three independent replicates with their corresponding standard deviation and significance (^**^ *p* < 0.01).

The overall odor intensity (ODI) of S-1 was rated as very strong, similar to S-4 (ODI = 7.8 and 6.6 respectively; *p* > 0.05). S-2 and S-3 had lower ODI scores (5.1 and 5.3 respectively; *p* > 0.05). Accordingly, the ODI of S-1 was rated significantly more intense than the ODI of S-2 and S-3 (*p* < 0.01; Figure 2).

### Total lipid and fatty acids

**Figure 3:**
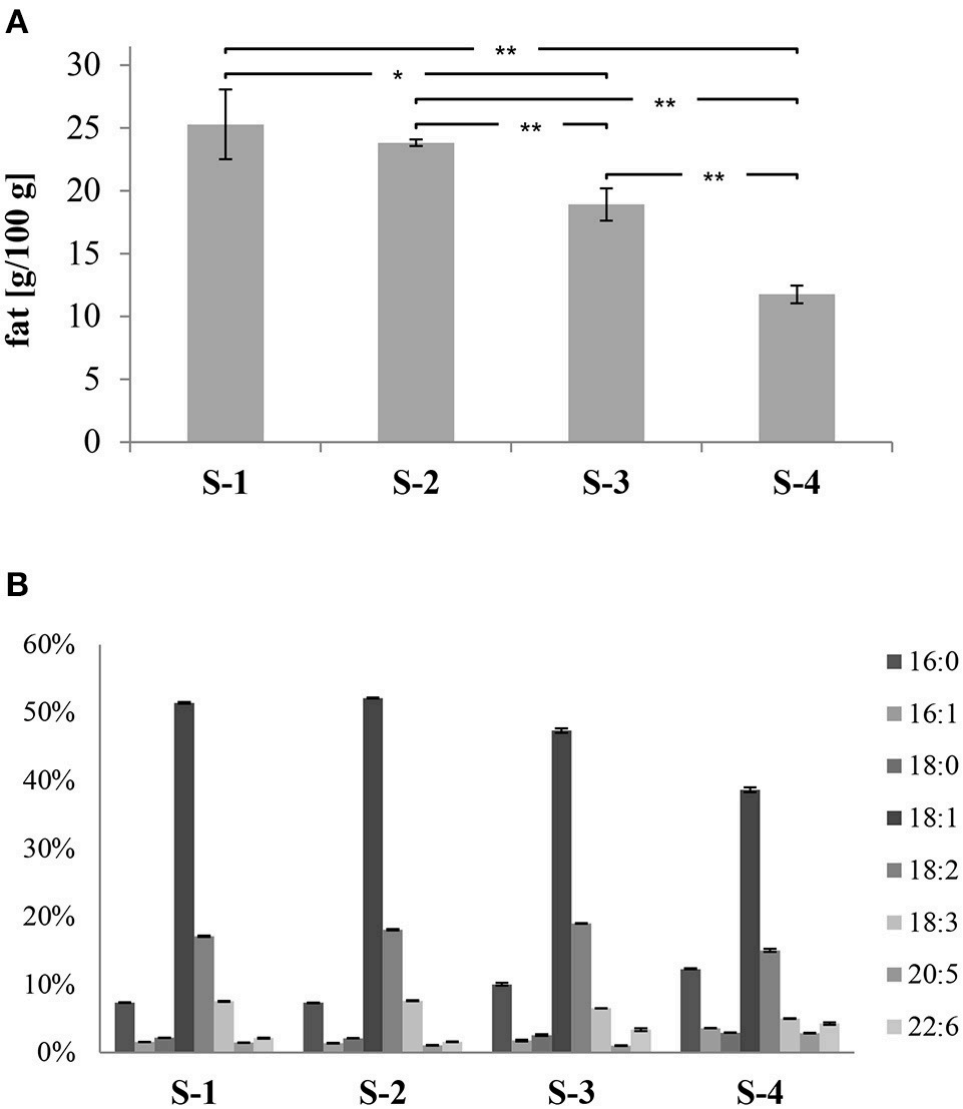
**(A)** Total fat content of samples (g/100 g); **(B)** percentages of the individual fatty acids of samples, shown as relative amounts of total fat. The data are displayed as mean values of three independent replicates with their corresponding standard deviation and significance (^**^p < 0.01 and ^*^ *p* < 0.05).

Based on the amount of total fat (TF), S-1 and S-2 had the highest values (25.3 ± 2.8 and 23.3 ± 0.3; g/100 g, respectively; see Figure 3A). On the other hand, S-4 showed the lowest value (11.8 ± 0.7; g/100 g) and S-3 scored in between (18.9 ± 1.3; g/100 g). Significant differences were found between the total fat contents of the samples, except for S-1 and S-2.

Based on the fatty acid (FA) contents, significant differences were found among samples, except between S-1 and S-2. In Figure 3B, the most common saturated fatty acids (SFA), monounsaturated fatty acids (MUFA), and polyunsaturated fatty acids (PUFA) are shown as relative amounts of TF (total fat). The S-4 feed scored the highest ΣSFA values (15%) followed by S-3 (12%), whereas S-1 and S-2 had equal values of 9%. Palmitic acid (16:0) was the most abundant SFA in all the samples. On the other hand, the S-4 feed had the lowest percentage of ΣEMUFA (43%), whereas higher percentages were found in S-3 (49%), and S-1 and S-2 (53% for both samples). Oleic (18:1*n* – 9) acid was the dominant MUFA in all the samples. Regarding the PUFA, eicosapentaenoic acid (20:5*n* – 3; EPA) and docosahexaenoic acid (22:6*n* – 3; DHA) are typical for fish feed. The highest value of the sum of these two PUFA was in S-4 (7%), then S-3 (4%), and finally both S-1 and S-2 had equal percentages of 3% (Figure 3).

Grigorakis et al. (2009) reported that fish oil-based formulas showed higher percentages of ΣSFA and EPA-DHA than plant oil-based formulas (30% higher ΣSFA and 67% higher EPA-DHA compared to the feeds with soy oil based-formula; 54% higher ΣSFA and 75% higher EPA-DHA compared to the rapeseed oil based-formula). Comparable results were reported by Baron et al. (2013). Thus, with increasing amounts of fish oil, the percentage of ΣSFA and EPA-DHA also increases. Consequently, the amount of the fish oil added to the samples was presumably in the following descending order: S-4, S-3, then S-1 and S-2 with similar amounts of fish oil.

### Aroma compounds

**Figure 4:**
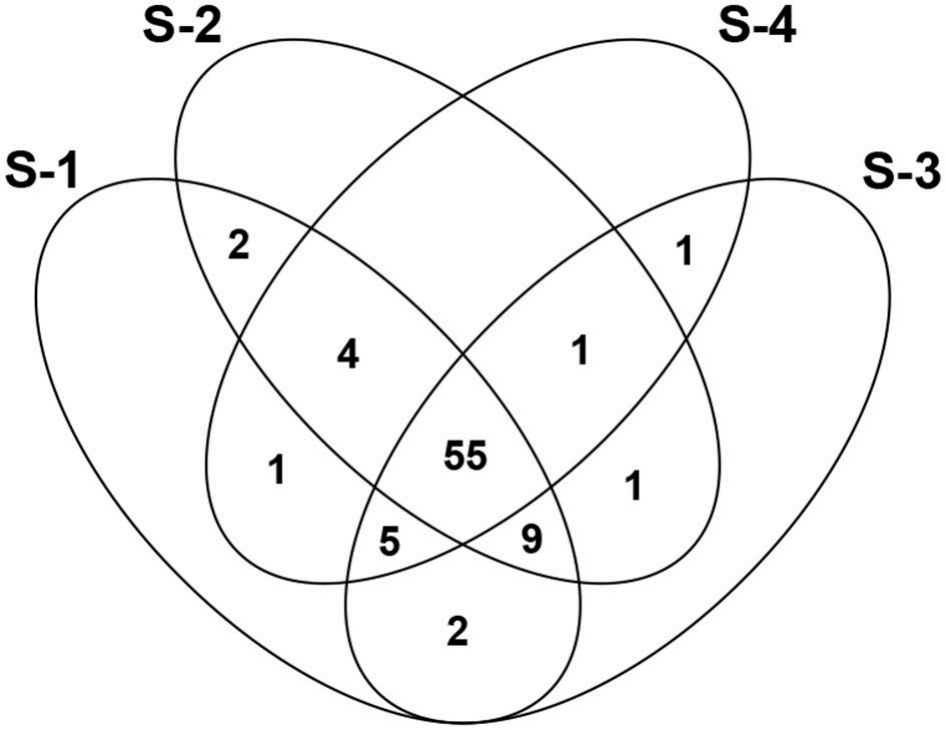
Venn diagram showing aroma compounds common to the samples.

A total of 81 compounds were olfactorily detected (Table 1). One of these compounds could not be identified due to its low concentration and hence lack of detection in MS analysis. AEDA was then performed to screen for the odorants detectable in the step-wise dilutions of the original distillate. 78, 72, 74, and 67 compounds were perceived in samples of S-1, S-2, S-3, and S-4 respectively. 55 compounds were common to the four samples, while 9 compounds were common to S-1, S-2, and S-3, 5 compounds to S-1, S-3, and S-4, 4 compounds to S-1, S-2, and S-4, 2 compounds to S-1 and S-2, 2 compounds to S-1 and S-3, 1 compound to S-2, S-3, and S-4, 1 compound to S-2 and S-3, and finally 1 compound was common to S-3 and S-4 (Figure 4).

**Table 1:** Potent aroma compounds identified by GC–O/MS.

**Label**	**Compound**	**Odor attribute^a^**	**RI**		**FD-factor^b^**				**Identification method^c^**
					**S-1**	**S-2**	**S-3**	**S-4**	
			**FFAP**	**DB-5**					
VOC1	2,3-Butanedione	Buttery, sweet	986	722	27	27	81	9	O, RI, MS, Std
VOC2	α-Pinene	Conifer-like	1013	940	N.D.	N.D.	81	27	O, RI, MS, Std
VOC3	α-Phellandrene	lemon-like, eucalyptus-like	1060	1000	9	27	27	9	O, RI, MS, Std
VOC4	Dimethyldisulfide	Sulfury, garlic-like	1070	780	N.D.	3	27	N.D.	O, RI, MS, Std
VOC5	Hexanal	Grassy	1078	800	3	27	81	3	O, RI, MS, Std
VOC6	(*Z*)-3-Hexenal	Grassy	1140	790	9	27	27	9	O, RI, MS, Std
VOC7	β-Myrcene	Metallic, geranium-like	1150	991	9	9	27	27	O, RI, MS, Std
VOC8	Cumene	Glue-like, petroleum-like	1166	925	243	9	729	9	O, RI, MS, Std
VOC9	Heptanal	Citrus-like, potato-like	1178	900	729	27	81	N.D.	O, RI, MS, Std
VOC10	D-Limonene	Lemon peel-like	1180	1025	729	3	27	27	O, RI, MS, Std
VOC11	(*Z*)-4-Heptanal	Fishy, fatty	1229	895	81	3	81	27	O, RI, MS, Std
VOC12	γ-terpinene	Citrus-like, green	1234	1063	81	27	234	27	O, RI, MS, Std
VOC13	*p*-Cymene	Oregano-like	1250	1039	27	3	27	N.D.	O, RI, MS, Std
VOC14	1-Octen-3-one	Mushroom-like	1270	985	9	81	9	9	O, RI, MS, Std
VOC15	Octanal	Citrus-like	1280	1005	9	81	27	9	O, RI, MS, Std
VOC16	Dimethyltrisulfide (DMTS)	Sulfury, garlic-like	1366	975	729	27	9	729	O, RI, MS, Std
VOC17	(*Z*)-1,5-octadien-3-one	Geranium-like, metallic	1367	982	9	243	N.D.	N.D.	O, RI, MS, Std
VOC18	Nonanal	Fatty, citrus-like	1380	1001	81	27	81	N.D.	O, RI, MS, Std
VOC19	Trimethylpyrazine	Earthy	1400	1022	243	9	729	N.D.	O, RI, MS, Std
VOC20	(*E*)-2-Octenal	Fatty, soapy, grassy	1415	1058	9	81	27	27	O, RI, MS, Std
VOC21	2-Ethyl-3,6-dimethylpyrazine	Pea-like	1425		81	N.D.	729	N.D.	O, RI, MS, Std
VOC22	Acetic acid	Vinegar-like	1440		81	81	9	27	O, RI, MS, Std
VOC23	Methional	Cooked potato-like	1445	310	2187	729	729	729	O, RI, MS, Std
VOC24	1-Octen-3-ol	Mushroom-like	1447	1000	27	2187	9	729	O, RI, MS, Std
VOC25	2-Ethyl-3,5-dimethylpyrazine	Moldy	1449	1099	243	243	729	243	O, RI, MS, Std
VOC26	3-Isopropyl-2-methoxypyrazine (IPMP)	Pea-like, green pepper-like	1450	1100	81	27	81	81	O, RI, MS, Std
VOC27	5-*sec*-Butyl-2,3-dimethylpyrazine	Pea-like, green pepper-like	1470		27	3	81	N.D.	O, RI, MS, Std
VOC28	2-Ethylhexan-1-ol	Musty	1481	1042	27	9	27	N.D.	O, RI, MS, Std
VOC29	Decanal	Citrus-like	1486	1233	81	27	243	81	O, RI, MS, Std
VOC30	(*E,E*)-2,4-Heptadienal	Fatty	1489	1020	81	27	729	243	O, RI, MS, Std
VOC31	(Z)-2-Nonenal	Fatty, cardboard-like	1490	1149	243	81	243	9	O, RI, MS, Std
VOC32	Propanoic acid	Fruity, cheesy	1500	880	243	243	9	81	O, RI, MS, Std
VOC33	(*E*)-2-Nonenal	Fatty, cardboard-like	1520	1160	243	243	2187	81	O, RI, MS, Std
VOC34	Linalool	Flowery, fresh	1530	1105	81	81	81	81	O, RI, MS, Std
VOC35	(*E,E*)-2,6-Nonadienal	Green, cucumber-like	1567	1153	729	27	N.D.	81	O, RI, MS, Std
VOC36	β-Caryophyllene	Earthy, green	1570	1436	243	27	81	729	O, RI, MS, Std
VOC37	(*E,Z*)-2,6-Nonadienal	Cucumber-like	1573	1160	243	243	27	243	O, RI, MS, Std
VOC38	Butanoic acid	Cheesy, sweaty	1605	800	243	243	234	81	O, RI, MS, Std
VOC39	(*E*)-2-Decenal	Fatty, coriander-like	1615	1261	729	81	81	729	O, RI, MS, Std
VOC40	(*E,E*)-2,4-Nonadienal	Fatty	1654	1213	81	81	234	243	O, RI, MS, Std
VOC41	2-/3-methylbutanoic acid	Sweaty	1666	860	243	243	729	81	O, RI, MS, Std
VOC42	(*E,Z*)-2,4-Nonadienal	Fatty, cucumber-like, cardboard-like	1690	1180	81	27	729	2187	O, RI, MS, Std
VOC43	Pentanoic acid	Fruity, sweaty, pungent	1720	890	2187	N.D.	27	729	O, RI, MS, Std
VOC44	(*E*)-2-Undecenal	Coriander-like, fatty	1745	1360	81	2187	234	2187	O, RI, MS, Std
VOC45	Geosmin	Earthy	1800	1413	27	9	729	N.D.	O, RI, Std
VOC46	(*E,E*)-2,4-Decadienal	Fatty	1805	1330	729	9	81	27	O, RI, MS, Std
VOC47	Hexanoic acid	Musty	1825	988	81	N.D.	27	27	O, RI, MS, Std
VOC48	Guaiacol	Smoky, vanilla-like	1845	1087	27	27	N.D.	729	O, RI, MS, Std
VOC49	Phenylethanol	Flowery, fresh	1900	1135	729	27	729	2187	O, RI, MS, Std
VOC50	ß-Ionone	Violet-like, flowery	1915	1490	729	81	729	243	O, RI, MS, Std
VOC51	Heptanoic acid	Sweaty, dusty	1930	1089	729	27	27	81	O, RI, MS, Std
VOC52	tr-4,5-Epoxy-(*E*)-2-decenal	Metallic	2000	1378	27	27	27	81	O, RI, MS, Std
VOC53	*o*-Cresol	Phenolic, ink-like, medical	2010	1063	243	3	81	N.D.	O, RI, MS, Std
VOC54	γ-Nonalactone	Coconut-like	2020	1368	27	81	81	243	O, RI, MS, Std
VOC55	Octanoic acid	Musty, coriander-like	2040	1179	243	27	27	729	O, RI, MS, Std
VOC56	δ-Nonalactone	Coconut-like	2050	1390	81	243	27	9	O, RI, MS, Std
VOC57	2-Ethylphenol	Fruity, sweet, ink-like	2059	1155	81	N.D.	N.D.	243	O, RI, MS, Std
VOC58	*p*-Cresol	Horse stable-like, fecal	2070	1087	729	243	729	81	O, RI, MS, Std
VOC59	*m*-Cresol	Leather-like, phenolic, ink-like	2088	1100	243	81	N.D.	2187	O, RI, MS, Std
VOC60	(*E,Z,Z*)-2,4,7-Tridecatrienal	Bloody, sweaty	2100	1577	9	27	27	81	O, RI, MS, Std
VOC61	2,3-/2,4-Dimethylphenol	Phenolic, leather-like	2100	1165	729	2187	27	N.D.	O, RI, MS, Std
VOC62	γ-Decalactone	Peach-like, fruity	2120	1478	27	N.D.	27	N.D.	O, RI, MS, Std
VOC63	Eugenol	Clove-like	2130	1350	3	81	N.D.	9	O, RI, MS, Std
VOC64	Nonanoic acid	Musty	2150	1276	81	9	27	27	O, RI, MS, Std
VOC65	4-Ethylphenol	Horse stable-like, fecal	2160	1178	2187	3	2187	729	O, RI, MS, Std
VOC66	4-Ethyloctanoic acid	Goat-like	2180	1338	729	N.D.	2187	2187	O, RI, Std
VOC67	δ-Decalactone	Coconut-like, sweet	2184	1510	N.D.	2187	27	81	O, RI, Std
VOC68	Sotolone	Maggi-like	2200	1108	81	N.D.	2178	81	O, RI, MS, Std
VOC69	Rotundone	Black pepper-like	2250	1715	27	2187	9	2187	O, RI, MS, Std
VOC70	Decanoic acid	Coriander-like, plastic-like	2259	1371	729	729	81	243	O, RI, MS, Std
VOC71	2-Methyldecanoic acid	Pungent, soapy, citrus-like	2278		2187	N.D.	27	2187	O, RI, Std
VOC72	Undecanoic acid	Coriander-like, fatty	2370	1487	81	81	27	81	O, RI, MS, Std
VOC73	γ-Dodecalactone	Peach-like, flowery	2379	1685	81	729	27	2178	O, RI, MS, Std
VOC74	Coumarin	Coconut-like, forest-like	2400	1440	81	27	81	81	O, RI, MS, Std
VOC75	Indole	Fecal, musty	2415	1278	729	243	729	729	O, RI, MS, Std
VOC76	Dodecanoic acid	Musty, plastic-like	2470	1560	729	81	27	27	O, RI, MS, Std
VOC77	Skatole	Fecal	2500	1390	729	2187	2187	2178	O, RI, MS, Std
VOC78	Ethylvanillin	Vanilla-like, smoky	2530	1475	243	27	N.D.	N.D.	O, RI, MS, Std
VOC79	Phenylacetic acid	Bee wax-like	2540	1250	2178	729	2187	2178	O, RI, MS, Std
VOC80	Vanillin	Vanilla-like	2550		729	729	2187	2178	O, RI, MS, Std
VOC81	Unknown	Mouth saliva-like	2750		243	2178	729	9	O, RI

*N.D.: not detected*.

a *Odor description as described by the panelists*.

b *FD-factors on FFAP column*.

c *Compounds were identified by: O, odor quality; RI, retention indices on DB5 and DB-FFAP columns; MS, mass spectrum (EI-mode), Std: comparison with reference*.

The major group of odor-active compounds was the aldehydes, which was mainly composed of fatty acid derived saturated and unsaturated compounds (17 identified aldehydes). The aldehydes with the highest dilution factors were vanillin (vanilla-like; FD = 729, 729, 2187, and 2178 in S-1, S-2, S-3, and S-4 respectively), (*E*)-2-undecenal (coriander-like, fatty; FD = 81, 2187, 234, and 2187 in S-1, S-2, S-3, and S-4 respectively), (*E,Z*)-2,4-nonadienal (fatty, cucumber-like, cardboard-like; FD = 81, 27, 729, and 2187 in S-1, S-2, S-3, and S-4respectively), (*E*)-2-nonenal (fatty, cardboard-like; FD = 243, 243, 2187, and 81 in S-1, S-2, S-3, and S-4 respectively), and (*E*)-2-decenal (fatty, coriander-like; FD = 729, 81, 81, and 729 in S-1, S-2, S-3, and S-4 respectively). (*E,Z,Z*)-2,4,7-Tridecatrienal showed lower FD values compared to the other aldehydes, though it has a very distinctive blood-like smell. This compound may increase in concentration when bloodmeal is used in the fish feed formula.

The high intensities of aldehydes in the four fish feed samples are in line with these having been reported as the main aroma compounds in aquaculture water, fish meat, fish oils, and meals (Selli et al., 2006, 2009; Mahmoud and Buettner, 2016, 2017; Salum et al., 2017), which might indicate that feeds are one of the primary enrichment sources of aldehydes in fish.

Amongst the 15 acids that were successfully identified, the most potent ones were phenylacetic acid (honey, bee wax-like; FD = 2178, 729, 2187, and 2178 in S-1, S-2, S-3, and S-4 respectively), 4-ethyloctanoic acid (goat-like; FD = 729, N.D., 2187, and 2187 in S-1, S-2, S-3, and S-4 respectively), 2-methyldecanoic acid (pungent, soapy, citrus-like; FD = 2187, N.D., 27, and 2187 in S-1, S-2, S-3, and S-4 respectively), and pentanoic acid (sweaty, pungent; FD = 2187, N.D., 27, and 729 in S-1, S-2, S-3, and S-4 respectively). As clearly indicated, most of them were associated with negative smell impressions by the panelists. Our previous studies have reported 4-ethyloctanoic acid in aquaculture water and related fish (Mahmoud and Buettner, 2016, 2017). At that time we could not disclose the source of such compounds under aquaculture conditions and proposed an exogenous accumulation scenario. Identification of this compound in feed samples could support its exogenous origin.

From the group of terpenes and terpene-related odorants, several compounds were detected with various intensities and smell characteristics: rotundone (black pepper-like; FD = 27, 2187, 9, and 2187 in S-1, S-2, S-3, and S-4 respectively), β-ionone (violet-like, flowery; FD = 729, 81, 729, and 243 in S-1, S-2, S-3, and S-4 respectively), β-caryophyllene (earthy, green; FD = 243, 27, 81, and 729 in S-1, S-2, S-3, and S-4 respectively), and cumene (glue-like, petroleum-like; FD = 243, 9, 729, and 9 in S-1, S-2, S-3, and S-4 respectively). Geosmin, the major cause of off-flavor in aquaculture was also detected (earthy; FD = 27, 9, 729, and N.D. in S-1, S-2, S-3, and S-4 respectively). The accumulation of malodor terpenes from feeds in fish was first proposed by Podduturi et al. (2017). The authors reported seven terpenes in their feed samples, including *p*-cymene, limonene, and α-pinene, which were also identified here. However, to the best of our knowledge, the other terpenes and terpene-related compounds identified in our study have not previously been reported in fish feed samples; this includes geosmin which might originate from fish oil. A reason for geosmin being present in fish oil might be that it had been extracted from fish that had previously accumulated geosmin during farming. The other terpenes might derive from plant raw materials used in feeds (Podduturi et al., 2017). The accumulation of terpenes in fish has previously been discussed in numerous reports (Selli et al., 2006, 2009; Selli and Cayhan, 2009; Cayhan and Selli, 2010; Podduturi et al., 2017).

Nine phenolic derivatives, cresols and indoles, were detected with very distinctive smells ranging from phenolic to fecal. The most potent smelling substances were skatole (fecal; FD = 729, 2187, 2187, and 2187 in S-1, S-2, S-3, and S-4 respectively) and 4-ethylphenol (horse stable-like, fecal; FD = 2187, 3, 2187, and 729 in S-1, S-2, S-3, and S-4 respectively). Part of these compounds is reported here for the first time as odor-active substances in fish feeds, including skatole, indole, and the cresols. Nevertheless, these compounds have previously been reported in aquaculture water and the related farmed fish (Farmer et al., 1995; Mahmoud and Buettner, 2016, 2017). The possible reasons for the presence of such compounds are the partial or total replacement of fish oil with lard and/or the usage of leftovers from slaughter material as raw material for feed (Zhou et al., 2015; Gerlach et al., 2016).

Five pyrazines were detected with moderate intensities. The most potent of these were: 2-ethyl-3,5-dimethylpyrazine (moldy; FD = 243, 243, 729, and 243 in S-1, S-2, S-3, and S-4 respectively) and trimethylpyrazine (earthy; FD = 243, 9, 729, and N.D. in S-1, S-2, S-3, and S-4 respectively). In our previous study, we also reported pyrazines in cultured fish and discussed that some of these pyrazines might result from a Maillard reaction occurring during feed processing (Mahmoud and Buettner, 2017). However, at that time, we could not prove this theory as no feed samples were analyzed. Our current study now confirms that fish feed could indeed be the source of such pyrazines. From these pyrazines, 3-isopropyl-2-methoxypyrazine is reported as a cyanobacterial by-product in aquaculture water; however it was identified in feed samples. Other cyanobacterial metabolites are dimethyldisulfide and dimethyltrisulfide (Ma et al., 2013). Still, these sulfur compounds were identified in feed samples. We believe that these odorants become enriched from the raw materials fish oil and/or fishmeal, meaning that these ingredients might have been prepared from fish that previously contained 3-isopropyl-2-methoxypyrazine, dimethyldisulfide, and dimethyltrisulfide. Nevertheless, the odor intensities of these compounds were relatively low and might be irrelevant in terms of the overall aroma impact. This needs to be confirmed by further studies.

Finally, several lactones, ketones, and alcohols were identified with smells varying from fruity and coconut-like (e.g., γ-nona- and γ-decalactone) to mushroom-like (e.g., 1-octen-3-one and−3–ol). Most of these compounds have hitherto not been reported in fish feed samples, but all these constituents have been reported in various cultured fish species (Selli et al., 2006; Selli and Cayhan, 2009; Mahmoud and Buettner, 2016, 2017).

Although all four fish feeds contained most of the compounds, there was also considerable variation among the samples. For instance, 2,3-/2,4-dimethylphenol revealed very high dilution factors in S-1 and S-2 (729 and 2187 respectively), whereas a low FD factor or even no smell detection was found in S-3 and S-4 (FD = 27 and ≤ 3 respectively). Another interesting example is 4-ethyloctanoic acid: this substance was detected with high dilution factors in S-1, S-3, and S-4, whereas it was absent in S-2. Supported by future quantitative experiments, such variations might shed light on the influence of diverse types of feed on the quantitative occurrence of specific odorants in fish and on potential off-flavor formation.

### Geometric projection of data using PCA

**Figure 5:**
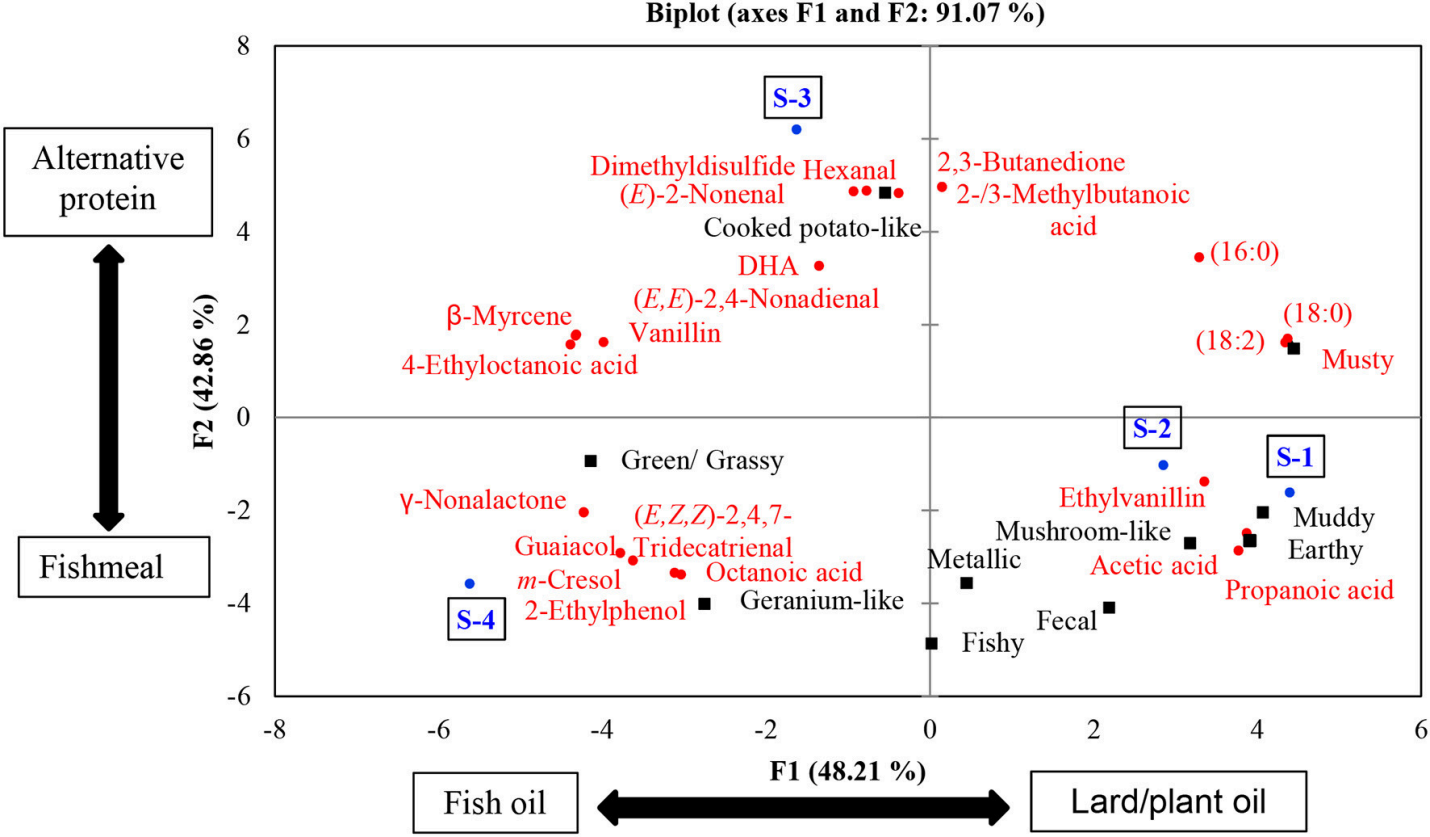
PCA bi-plot of odorants and fatty acid analyses showing two principal components that explain 91.07% of the variation. The blue color represents samples, the black color represents sensory attributes, and the red color represents chemical variables.

Principle component analysis was used to understand the variations among the samples. We used only the chemical variables that were significantly correlated with intensity ratings of sensory attributes for the PCA. These variables were 18 odorants and 4 fatty acids, all of which are named in Figure 5. Two principal components explained 91.07% of the variation. The first principal component (PC1) successfully differentiated between sample S-4 and the other samples. The second principal component (PC2) distinguished between S-3 and the other samples. This is reflected by the positioning of samples S-3 and S-4 on the chart, with each of them being in separate quadrants (S-4 in quadrant – PC1/ – PC2 and S-3 in quadrant – PC1/ + PC2). Weaker differences were observed between S-1 and S-2, being grouped in the quadrant + PC1/ – PC2. In other words, S-4 and S-3 showed differences in composition between each other, but also compared to the rest of the samples. On the other hand, S-1 and S-2 were ranked with similar compositions, but differed from both S-4 and S-3. The overall findings are discussed in more detail below.

**Figure 6:**
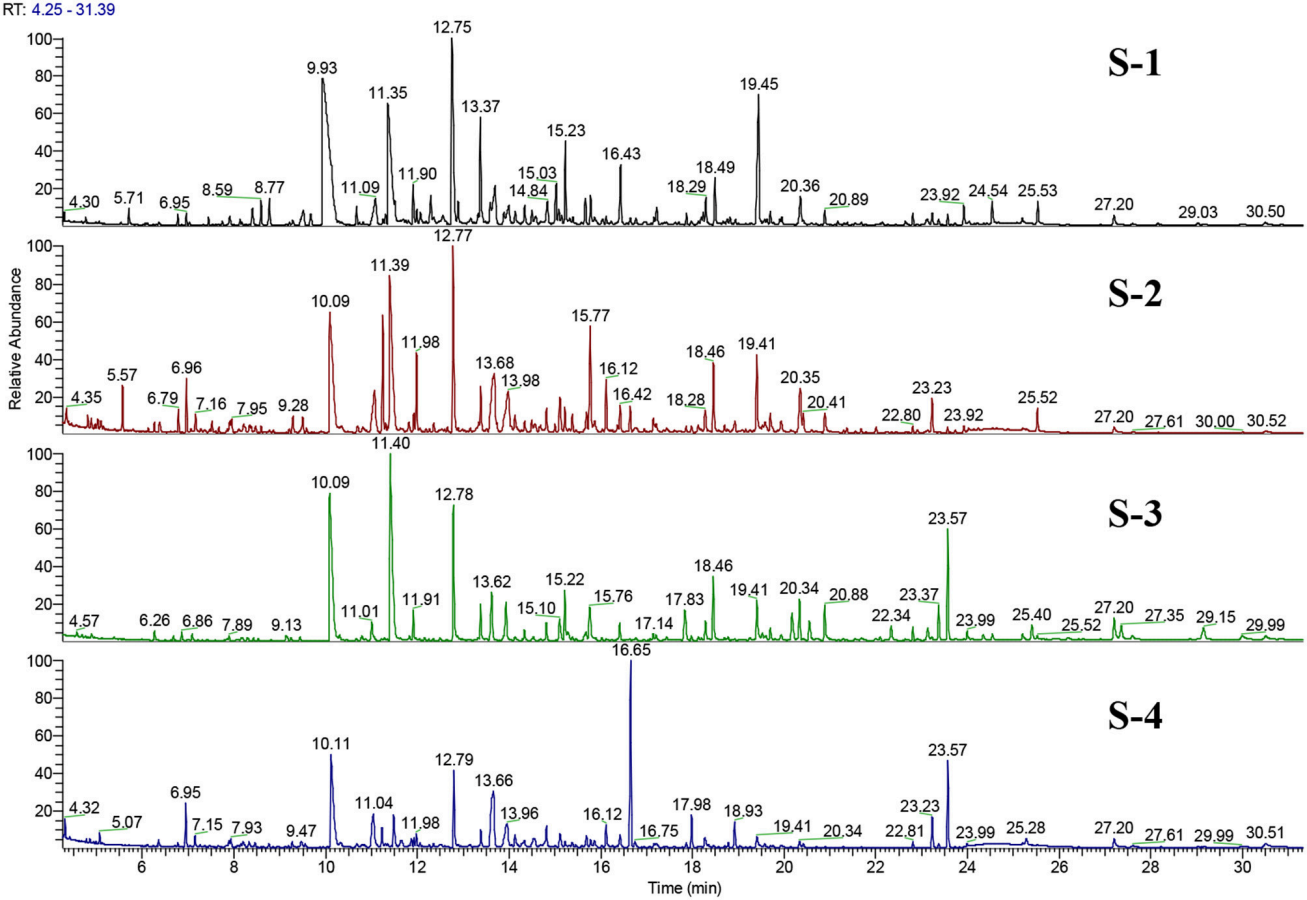
Total ion chromatogram (TIC) of GC-O/MS (EI-mode) representing separation of the volatile fraction of the four feed extracts. Both S-1 and S-2 exhibit the same major peaks, but in some cases with different intensities.

S-1 and S-2 exhibited no significant differences in their FA profile and TF content and had similar odorant profiles. Additionally, their TIC showed the same major peaks (see Figure 6). These findings correlate with their joint placement within the same PC quadrant. S-3 and S-4 on the other hand were significantly different in terms of their FA and TF contents.

Regarding the ODI scores and TF contents, S-1 had the highest values. Furthermore, S-1 had lower EPA and DHA contents than the S-3 and S-4 samples. At first sight this might indicate that the TF content is related to the odor intensity of the fish feed. However, S-2 had a similar TF content and ΣPUFA to S-1; accordingly, its ODI score was significantly lower than that of S-1. This means, in our opinion, that the ODI in S-1 is not only attributed to the lipid but also to the protein source. The study of Baron et al. (2013) on the FA profile of fish feeds having different formulas (fishmeal−fish oil, plant protein−fish oil, and plant protein−different plant oils) supports this assumption. No major differences in FA content were found between the fish oil-based formulas that contained different protein sources (plant protein or fishmeal). On the other hand, plant protein-based formulas that were produced from different oil sources (fish oil or plant oils) showed significant differences in the FA profile of the feed. Given that S-1 and S-2 exhibited similar TF and FA contents, the change in ODI score might be related to the higher fishmeal content in S-1. It is known that fishmeal has a more distinctive smell than plant protein, as reflected in feeds that contain higher amounts of fishmeal (Giogios et al., 2009; Baron et al., 2013).

A similar pattern was observed when S-3 was compared to S-4. However, this time S-4 can be expected to contain more fishmeal than S-3 as might be indicated by the positioning of S-4 and S-3 in the PCA grouping. In this context, S-4 contained the lowest level of TF. However, the ODI was comparable to the S-1 sample. Given that S-4 contained the highest levels of EPA and DHA, it is likely that the fishmeal and fish oil levels were higher in this product than in the other feeds. To conclude, we assume that PC1 differs mainly with regard to the lipid sources used in the formula (lard/plant oil in + PC1 and fish oil – PC1), and PC2 is divergent with regard to the protein sources used in the formula (fish meal in – PC2 and alternative protein sources in + PC2).

## Conclusion

The current study investigated whether fish feeds can be a potential source of off-odors in aquaculture water and fish. Comparative evaluation of the most commonly used fish feeds in Bavaria was performed based on their aroma profiles and lipid contents. Our findings confirmed the potential impact of fish feeds on off-odor accumulation in cultured fish. We showed that compounds such as skatole, 4-ethyloctanoic acid, indole, and cresols may originate from fish feeds. It is also suggested that feeds might be the primary source of fatty acid-derived volatile aldehydes that have previously reported in aquaculture water and fish. These odorants are not linked to the total fat content of the feeds, but rather to the amount of unsaturated fatty acids contained in the formula. The protein type (plant-based or fishmeal-based sources), in addition to the lipid content, is another factor that might play a significant role in odor formation. There might be a correlation between the protein type and the total aroma intensity of the samples. Based on our findings, feeds can now also be suspected as being the potential sources of compounds that have previously been thought of as being exclusively accumulated from cyanobacteria, for example geosmin and 3-isopropyl-2-methoxypyrazine. These findings call for further investigations on the complex mechanisms of off-flavor formation in aquacultural systems and fish with the aim of improving the quality of aquaculture products.

## Author contributions

Each author participated sufficiently in the work, intellectually or practically, to take public responsibility for the content of this article, including the conception, design, and conduct of the experiment and data analysis and interpretation. AB, MM, and TT participated in the design of the study. MM and TT were responsible for sampling. MM carried out the chemo-sensory analysis and helped TT with lipid analysis. AB, MM, TT, HL, and MW conceived the study. MM was responsible for data analysis and wrote the paper. All authors contributed to the manuscript and approved the final version.

### Conflict of interest statement

The authors declare that the research was conducted in the absence of any commercial or financial relationships that could be construed as a potential conflict of interest.
